# Nitrate in groundwater and agricultural products: intake and risk assessment in northeastern Iran

**DOI:** 10.1007/s11356-022-20831-9

**Published:** 2022-06-13

**Authors:** Mohammad Zendehbad, Majid Mostaghelchi, Mohsen Mojganfar, Peter Cepuder, Willibald Loiskandl

**Affiliations:** 1 University of Natural Resources and Life Sciences, Vienna, Department of Water, Atmosphere and Environment, Institute of Soil Physics and Rural Water Management, Muthgasse 18, 1190 Vienna, Austria; 2 University of Vienna, Faculty of Geosciences, Geography and Astronomy, Department of Mineralogy and Crystallography, UZA 2, Althanstraße 14, 1090, Vienna, Austria; 3 Ferdowsi University of Mashhad, Faculty of Science, Department of Geology, Azadi Square, Mashhad, Iran

**Keywords:** Nitrate, Groundwater, Health risk assessment, Fruits and vegetables, Drinking water, Water quality

## Abstract

The suitability of groundwater and agricultural products for human consumption requires determining levels and assessing the health risks associated with potential pollutants. Here, particularly pollution with nitrate still remains a challenge, especially for those urban areas suffering from insufficient sewage collection systems, resulting in contaminating soil, endangering food safety, and deteriorating drinking water quality. In the present study, nitrate concentrations in the commonly consumed fruit and vegetable species were determined, and the results, together with the groundwater nitrate levels, were used to assess the associated health risks for Mashhad city residents. For this assessment, 261 water samples and 16 produce types were used to compute the daily intake of nitrate. Nitrate in groundwater was analyzed using a spectrophotometer, and produce species were examined using High-Performance Liquid Chromatography. Ward’s hierarchical cluster analysis was applied for categorizing produce samples with regard to their nitrate content. Additionally, to account for the sanitation hazards associated with groundwater quality for drinking purposes, total coliform and turbidity were also assessed using the membrane filter (MF) technique and a nephelometer, respectively. Nitrate concentrations exceeded the prescribed permissible limits in 42% of the groundwater wells. The outcomes also exhibit significantly higher nitrate accumulation levels in root-tuber vegetables and leafy vegetables compared to fruit vegetables and fruits. Using cluster analysis, the accumulation of nitrate in vegetables and fruits was categorized into four clusters, specifying that radish contributes to 65.8% of the total content of nitrate in all samples. The Estimated Daily Intake (EDI) of nitrate and Health Risk Index (HRI) associated with consumption of groundwater exceeded the prescribed limit for the children’s target group in Mashhad’s south and central parts. Likewise, EDI and HRI values for produce consumption, in most samples, were found to be in the tolerable range, except for radish, lettuce, and cabbage, potentially posing risks for both children and adult consumers. The total coliforms in groundwater were found to violate the prescribed limit at 78.93% of the sampling locations and were generally much higher over the city’s central and southern areas. A relatively strong correlation (*R*^2^ = 0.6307) between total coliform and nitrate concentrations suggests the release of anthropogenic pollution (i.e., sewage and manure) in the central and southern Mashhad.

## Introduction

Over the last decades, the rapidly growing trend of urbanization, alongside the obligate agricultural/industrial developments, has inflicted multiple contaminants of urgent concern on the environment (Mostaghelchi et al. 2021). Due to the broad dispersion into the soil and high solubility in water, nitrate is likely the most globally widespread contaminant, posing a detrimental influence on the quality of drinking water resources and agricultural products. It naturally occurs in the soil through the microbial transformation of ammonia under oxic conditions which is released by organic compounds such as organic fertilizer, plant and waste decomposition or by mineral fertilizers. Nitrate is not chemically bound to soil components, making it predisposed to leach through the soil and into groundwater and thus impacting both surface and groundwater quality. The nitrate concentration in groundwater, vegetables, and fruits has been observed to exceed the acceptable levels worldwide as well as in many parts of Iran (Gao et al. 2012; Pastén-Zapata et al. 2014; Bahadoran et al. 2016; Qasemi et al. 2018; Mehri et al. 2019; Zendehbad et al. 2019a; Boumaiza et al. 2020). In urban environments, several anthropogenic factors can be considered as the main sources of nitrate contamination in surface and ground waters, including leakage from sewer collection systems, discharge of industrial effluents and municipal wastewater, landfills, and usage of fertilizers and manure in urban green belts and urban/peri-urban agriculture (Wakida and Lerner, 2005; Zendehbad et al. 2019a). Additionally, septic system leakage and organic matter decomposition can also cause total coliforms to increase in groundwater resources (Ling, 2000; Smoroń, 2016; Mititelu-Ionuș et al. 2019).

Nitrate pollution has become an increasingly critical issue in Iran, where groundwater is the primary source of agricultural, domestic, and drinking water. The nitrate concentrations in groundwater resources of different parts of Iran reported in recent studies are summarized in Table 1. Mashhad is an exemplary case, where its urban groundwater suffers from contamination issues (Zendehbad et al. 2019b). Old wastewater disposal and septic systems, discharging a high level of nitrogen into the groundwater, were found to be the primary source of nitrate pollution in Mashhad urban groundwater (Zendehbad et al. 2019a). In such places where groundwater is the main water supply for both drinking and agricultural purposes, besides potentially threatening food safety, the excessive nitrate content in drinking water may cause considerable health risks to the residents.

**Table 1:** Reported nitrate concentrations for groundwater from different parts of Iran

Nitrate range (mg/L)	Study area (province)	Reference
3.20–428.00	Kurdistan	(Solgi and Jalili, 2021)
0.5–225	Kurdistan	(Maleki and Jari, 2021)
2.43–96	Fars	(Bahrami et al. 2020)
3.09–88.5	Kermanshah	(Jalili et al. 2018)
1.7–155.5	Alborz - Azarbaijan	(Barzegar et al. 2017)
3.05–181.7	Alborz	(Chitsazan et al. 2017)
11.5–148.7	West Azarbaijan	(Esmaeili et al. 2018)
12.8–203	Tehran	(Ghahremanzadeh et al. 2018)
0.1–218	Mazandaran	(Rasuli et al. 2018)
8–166.1	Razavi Khorasan	(Zendehbad et al. 2019a)

Drinking water is not considered as the major source of nitrate intake when the concentration is low. However, by exceeding the World Health Organization (WHO) standard of 50 mg/L (World Health Organization, 2017), it becomes potentially the primary source of intake, unless other high nitrate intake sources such as fruits and vegetables are present (Fan, 2011). Several studies confirmed that fruits and vegetables contain high nitrate content and account for approximately 85% of the dietary nitrate intake across many communities (Bahadoran et al. 2016; Bondonno et al. 2018; Chetty et al. 2019). Among several contamination factors, such as irrigation with untreated wastewaters and pesticide residue that may lead to nitrate accumulation in fruits and vegetables, the excessive application of nitrogenous fertilizers is considered the most critical factor, which has arisen as an alarming public health concern (Fan, 2011). The nitrate accumulation levels in fruits and vegetables also depend on several environmental, nutritional, and physiological factors that can vary from region to region, such as location, soil properties, crop’s biological properties, humidity, light intensity, and daytime temperature, as well as the method of cultivation, crop rotation, vegetation period, the season of harvest, and fertilization (Correia et al. 2010; Parks et al. 2012). Several vegetables (e.g., radish, spinach, lettuce, broccoli, beetroot, celery, and cabbage) were shown to have an augmented nitrate content of >1000 mg/kg. The concentration of nitrate in green leafy vegetables has been observed to be higher (Thomson et al. 2007). Higher nitrate accumulation in leafy vegetables, including spinach and lettuce, and several root crops such as beetroot and radish, may be associated with the accumulation tendency of higher nitrate concentrations in the plant’s leaves as well as stems or roots. Accordingly, nitrate has been usually observed at lower levels in fruity vegetables (or flowers) and fruits (FSAN, 2011).

Still, fruits and vegetables are believed to have a preventing role in health risks caused by micronutrient deficiencies (e.g., cardiovascular disease, cancer, and mortality) (Bahadoran et al. 2016; Bondonno et al. 2018; Chetty et al. 2019). Thus, the WHO and Food and Agriculture Organization (FAO) encourage consuming a minimum of 400 g of fruits and vegetables per day (FAO/WHO, 2005). However, by failing to approximately 200 g/day, Iran’s fruits and vegetables per-capita usage are much lower and is half of WHO norms (Esteghamati et al. 2012).

Nitrate at high concentrations, however, may cause health risks, such as methemoglobinemia (in infants) (Tate and Arnold, 1990); diabetes (Kostraba et al. 1992), and can be a potential contributor to developing chronic diseases (Lundberg et al. 2008) and generating some cancer types in the human body (Chiu et al. 2007). Nitrate converts into nitrite in the human digestive system, which readily combines with the secondary amines and forms the N-nitroso compounds (nitrosamines). These compounds are firmly believed to be carcinogenic (Cross et al. 2011; Loh et al. 2011; Roohparvar et al. 2018). N-nitroso compounds have also increased cancer risk in laboratory animals in different studies (Zhu et al. 2014; Espejo-Herrera et al. 2015). While previous reports have confirmed the positive correlations between acute exposure to excessive nitrate concentrations and certain cancer types, including stomach, esophageal and gastric cancers (Yang et al. 1998; Nowrouz et al. 2012), some reports in contrast suggest potential preventive effects and health benefits of nitrate, including cardiovascular effects (e.g., blood-pressure-lowering effects) (Machha and Schechter, 2012; Kapil et al. 2015; Jonvik et al. 2016; Velmurugan et al. 2016; Kerley et al. 2018; Jackson et al. 2019). Other studies noticed that the formation of nitrosamines could be decreased with the high content of polyphenol and vitamin C in some fruits and vegetables by facilitating the non-enzymatic reduction of toxic nitrite to beneficial nitric oxide (Rocha et al. 2009; Erkekoglu and Baydar, 2010). However, the risk-benefit aspects of the dietary intake of nitrate are yet to be investigated, particularly in the places where fruits and vegetables are not the only sources of nitrate intake for the residents.

Due to the global concerns on water quality, multiple studies investigated the health risks of exposure to different pollutants through drinking water, both worldwide and in many parts of Iran (Mortada and Shokeir, 2018; Moeini and Azhdarpoor, 2021; Badeenezhad et al. 2021a, b; Mohammadpour et al. 2022). In Iran, Moeini and Azhdarpoor (2021) reported that the health risks for Shiraz residents were higher than the threshold values in those locations with the groundwater nitrate concentration exceeding the WHO standard, potentially posing a higher risk to infants and children. Badeenezhad et al. (2021b) also found an increasing trend in the maximum nitrate levels of Shiraz groundwater between 2013 and 2017, and consequently, health risks of nitrate exposure, particularly for the children’s age group, were also estimated to be increasing. The researchers reported that land-use changes in the study area, predominantly urban and residential developments, significantly affected the groundwater nitrate concentration and the degree of the associated health. In another study, children were found to be the significant at-risk group due to exposure to nitrate in drinking water in Behbahan (Badeenezhad et al. 2021a). Mortada and Shokeir (2018) found that nitrate levels in water samples of Mansoura city, Egypt, were within the acceptable limit, and the population’s health risk in these areas was also low. However, further studies on other potential sources of nitrate, such as fruits and vegetables, were recommended to provide a complete profile of the possible impacts.

Given the health risks associated with nitrate intake from fruits and vegetables, regulatory authorities have established the tolerable limit of dietary nitrate termed: Acceptable Daily Intake (ADI). The Scientific Committee for Food (SCF) of the European Commission (EC) stipulated the nitrate ADI of 3.7 mg nitrate/kg body weight/day. (ECETOC, 1988). Later, in 2002, the Joint WHO/FAO Expert Committee on Food Additives (JECFA) reaffirmed the limit for nitrate ADI (EFSA, 2008).

In the pretext of the above-mentioned health hazards and pollution pressures on water supply sources in Mashhad, where the population relies on groundwater for both drinking and agriculture, assessing nitrate health risks using different intake sources (i.e., water, fruits, and vegetables) is of great significance. The current status of nitrate in the Mashhad urban area remains unexplored, with limited investigations giving way to conducting further studies on the aspect. Therefore, the present study aims to determine the nitrate intakes and associated health risks with the consumption of both groundwater and agricultural products in Mashhad, using a high-resolution groundwater nitrate database as well as the measured nitrate concentrations of locally cultivated fruits and vegetables. The health risk evaluation was carried out based upon the established safety limits, Health Risk Index (HRI), and Acceptable Daily Intake (ADI). The outcomes are expected to assist the health professionals, decision-makers, and authorities devise better-detailed health management plans in terms of local and regional produce and drinking water quality protection.

## Materials and methods

### Description of study area

**Fig. 1:**
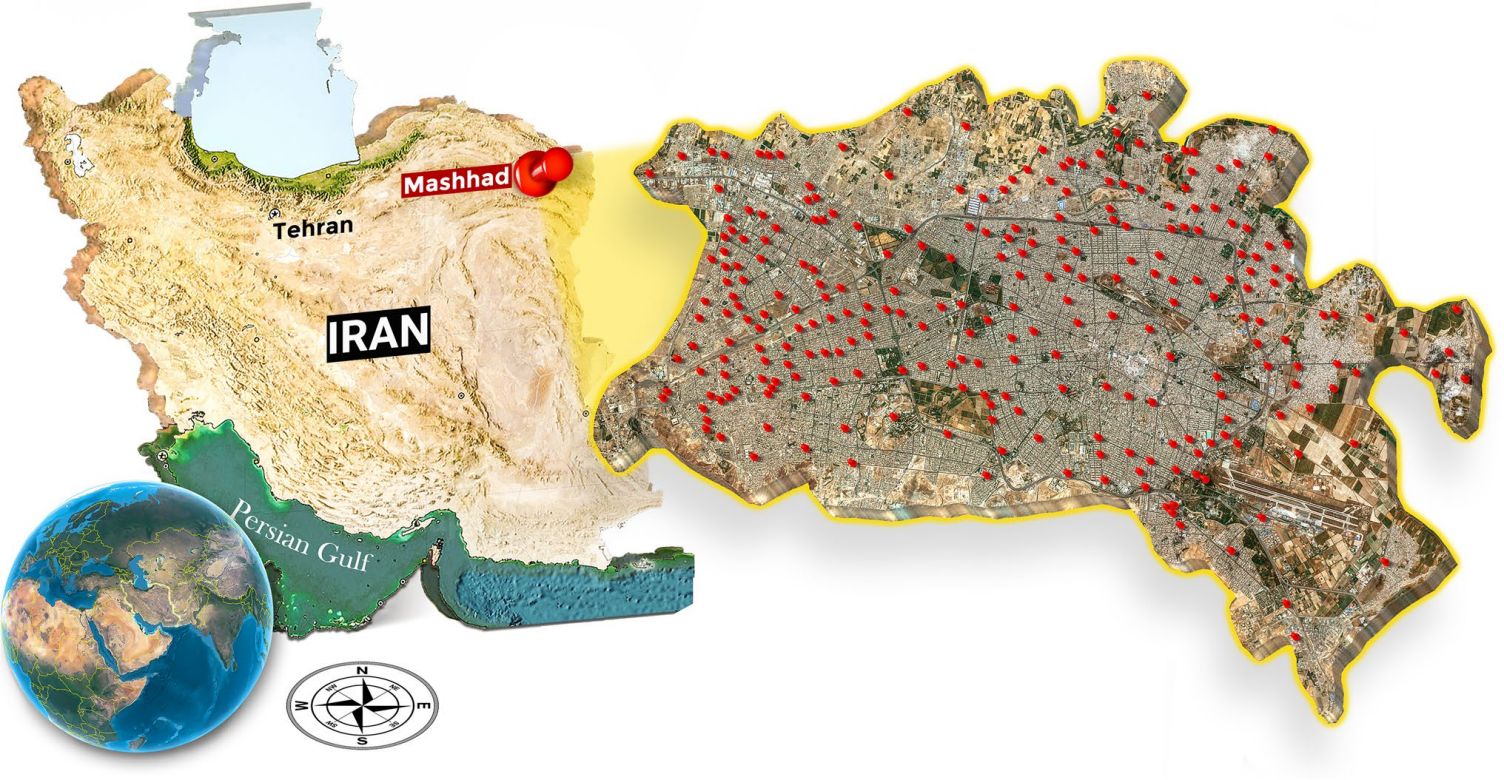
Location of the study area: Mashhad city, northeastern Iran; the red points represent sampling locations for groundwater

The study area is Mashhad — the second most populous city in Iran — situating in the North East of the country (between 36°.15′–36°.20′ N and 59°.30′–59°.40′ E) (Fig. 1). The city covers 280 km^2^ and has a population of larger than 3 million, with around 20 million tourists visiting annually. Locating in a semiarid climate, Mashhad region has wet-cold winters and dry-hot summers, with an average temperature of 13.5 °C (min −21 °C, and max 44 °C) and average annual precipitation of 253 mm (Khalili et al. 2016).

The geology of the area is comprised of various sedimentary rocks (e.g., marl, limestone, and clastic rocks) in northern margins and granitic, metamorphic, and ultrabasic outcrops (e.g., slate, phyllite, and schist) in the southern and southwestern parts. The city is positioned on thick deposits of Quaternary, and the Mashhad’s underneath sediments are a combination of ultramafic and metamorphic rocks (Zendehbad et al. 2019a). The aquifer of Mashhad is characterized as unconfined and composed of alluvial gravel-sand sediments of the Quaternary period. Groundwater flows according to the plain general slope, from northwest to southeast, and the industrial estate is located upstream. Approximately 220 million m^3^ of water is required to meet the yearly domestic demand, mainly supplied by the city’s groundwater (Zendehbad et al. 2019a). Mashhad’s average water usage is over 550,000 m^3^/d, and it is estimated that nearly 75% of the water (on a yearly basis) returns to groundwater (Ehteshami et al. 2014). Therefore, municipal return flow is the primary source of groundwater recharge. Southern and central areas — the city’s major old core with a higher density of population — suffer from an insufficient sewer collection system compared to the western part, which is newly developed as a result of the city’s expansion.

Mashhad groundwater is also being heavily pumped to meet agricultural water needs, significantly contributing to the area’s groundwater table declination (1.2 m/year). The area’s most common types of crops are cereals (55%), vegetables (21%), orchards (19%), and industrial crops (5%). Mashhad also grows a variety of fruits throughout the year. The gardens embedded in the dense urban fabric take on great relevance to the production of agricultural products. Alandasht garden (9.6 ha, in the city’s central part), Malek Abad garden (299 ha, in the city’s central part), Imam Reza garden (113 ha, in the city’s northern part), Astan-e-Quds gardens (771 ha, in the city’s northern part) are being used for urban agriculture and helping meet the need for local food. Private farms in the suburbs of Mashhad also play a significant role in response to the daily demand of consumers within the city. In recent decades, yields of leafy vegetables (such as lettuce) have increased (82%), and due to vegetable farmers’ use of untreated wastewater for irrigation, contamination of soil and crops (e.g., non-acceptable pathogen levels in leafy vegetables) has also increased (Danso et al. 2018).

### Sampling

#### ***Water***

The nitrate data presented in this study were obtained from a dataset with a high spatial resolution of groundwater wells from a previous study (Zendehbad et al. 2019a). The nitrate levels were determined using water samples collected from 261 groundwater wells. Although the area of study is situated in a semiarid climate, the quality of groundwater was purposely studied in a dry season to avoid water quality fluctuations resulting from potential short-term recharge in Mashhad’s wet season. Prior to the sampling, the wells were purged multiple times to ensure that the samples truly represent the formation of the aquifer. These wells provide the supply of the city’s water for drinking and irrigation needs. Information on the screening length and actual depth of these old drinking water wells is limited as they were mainly installed decades ago. The aquifer from which all the samples were collected was the same. Figure 1 displays sampling locations, covering the entire city. A submersible pump was used to collect groundwater samples around 3 to 5 m beneath the groundwater table. Turbidity measurements were conducted in situ using a turbidimeter (nephelometer). A 0.45 m membrane filter was used to filter the samples before pouring them into 60 ml PET bottles. The samples were stored at 0–4 °C and, quickly after collection, transferred to the lab.

#### ***Fruits and vegetables***

The fruit and vegetable species were chosen based upon two assumptions: (i) their consumption rate among the residents, and (ii) the nitrate content in vegetables and fruits grown in the region. Due to the different socio-demographic status of the residents and differences in the quality of supplied food items in the different areas, Mashhad was divided into four zones, i.e., south/central, north, west, and east. A random sample of 250 residents aged 20 years and over were requested to respond to a face-to-face survey specifying their household frequent fruit and vegetable items, and 222 subjects agreed. All the samples were randomly collected based on the locals’ and agricultural officers’ opinions and by considering their biological maturity from the main municipal markets and produce shops in each city’s specified area. The samples were also divided into four groups: (i) fruits (banana, apple, grape, orange), (ii) fruit vegetables (tomato, cucumber, cauliflower, eggplant, green bean, green pea, zucchini), (iii) tuber and root vegetables (carrot, potato, radish), and (iv) leafy vegetables (lettuce, cabbage). They were afterward placed into ziplock bags and, after being labeled, transferred to the lab. The prepared samples were stored at below 4 °C until the following day for analysis.

### Standard and reagent solutions

Potassium nitrate and hydrochloric acid, all in the analytical grade, and 1-Pentanesulfonic acid sodium salt and methanol, in HPLC grade, were purchased from Merck, Germany. For the preparation of the solutions as well as extraction of the samples, deionized water was used.

### Sample preparation

Vegetable and fruit samples from two different locations in each specified area of the city with three replicates were used for analysis. Preparation and extraction of the samples were carried out in accordance with the recommendations by the EC’s Directive 1882/2006 (Commission, 2006). Following the gathering of the samples, non-edible parts were discarded, and subsequently, the samples were cut into smaller pieces and thoroughly homogenized. Directly after, the samples were kept at –15 °C, waiting for the laboratory analysis. The homogenized samples (2 g) were placed into a 100 mL Erlenmeyer flask, and afterward, about 50 mL of deionized water was added. Next, it was kept in a boiling water bath (80 °C) for 15 min, shaken, and left undisturbed to cool down before diluting with deionized water to a final volume of 100 mL. Finally, after discarding the first 2 mL of the filtrate (using a 0.45–l m pore-sized filter), the solution was stored at 4 °C for nitrate determination analysis.

### Nitrate determination

Nitrate in drinking water samples was determined using a colorimetric method with a UV-visible spectrophotometer measured at 410 nm (Rowell, 2014). Measurement of nitrate in fruits and vegetables was conducted employing a High-Performance Liquid Chromatography (HPLC) (Waters, Milford, USA) equipped with an autosampler (Waters, model 717 plus, Milford, USA) and a photo array detector (Waters, model 996, Milford, USA) and the separation was carried out on a C_18_ column (4.6 mm × 250 mm, 5 μm; Waters, Ireland).

With certain adjustments, the nitrate in this investigation was evaluated using the method reported by Hsu et al. (2009). A buffer solution consisting of 2 g of 1-Pentanesulfonic acid sodium salt dissolved into 950 mL of deionized water, using a 1-L beaker, was used for analytical procedures. Potassium nitrate (160 mg) was also mixed into deionized water inside a volumetric flask to provide a range between 100 and 500 ppm of standard nitrate solutions for the preparation of the nitrate calibration curve. The obtained calibration curve from plotting the concentration (ppm) against the peak area exhibited satisfactory linearity (*R*^2^ ≥ 0.997), and the peak’s Relative Standard Deviation appeared to be less than 1%. Thus, in order to determine the nitrate content in chosen samples of vegetables and fruits, we used the calibration curve and expressed the values in mg/kg.

The mobile phase, which contained a solution of buffer and methanol (70% and 30%, respectively), was set to flow through the column of HPLC until a stable baseline signal was equilibrated. The mobile phase’s pH was adjusted with 2 M of hydrochloric acid to receive pH 3. The flow rate was 1 mL/min, and the column temperature was maintained at 40°C. The injection volume was 10 μL with a run time of 10 min, and the detection wavelength was set at 225 nm. Finally, the sample’s peaks were identified by comparing their retention times and peak areas with those of standard solutions.

### Turbidity and coliform determination

So as to account for the sanitation hazards attributed to sustaining the sufficient quality of water for drinking purposes, further parameters, i.e., evaluation of turbidity and coliform, were also incorporated in this research. According to the American Public Health Association’s (APHA) established standard methods (9222 D/B method), the membrane filter (MF) technique was employed to identify and estimate total coliforms. Turbidity measurements were performed employing a nephelometer (Eutech^TM^) as per method 2130 of APHA and expressed in Nephelometric Turbidity Units (NTU) (APHA, 2012).

### Exposure estimation

The intake of nitrate from water, vegetables, and fruits over the toxicity level or Acceptable Daily Intake (ADI) limit (3.7 mg nitrate/kg body weight (EFSA, 2008)) can cause health issues or even fatality (Du et al. 2007). The daily nitrate intake was calculated to estimate the average daily accumulation of nitrate in a consumer’s body of specific bodyweight and also to estimate the relative bioavailability of nitrate. It was separately determined for water and the samples of fruits and vegetables using the following Estimated Daily Intake (EDI) equation (Equation (1)), taking into account that the EDI represents only the possible ingestion rate and does not consider the potential metabolic excretion of nitrate (USEPA, 2004).

1 $$EDI=\left( AC\times C\right)/ BW$$

where AC stands for the average consumption of water per capita (L), and fruits and vegetables (g); C represents the nitrate concentration in water (mg/L), and fruits and vegetables (mg/kg); and, BW is the mean bodyweight of the consumer.

**Fig. 2:**
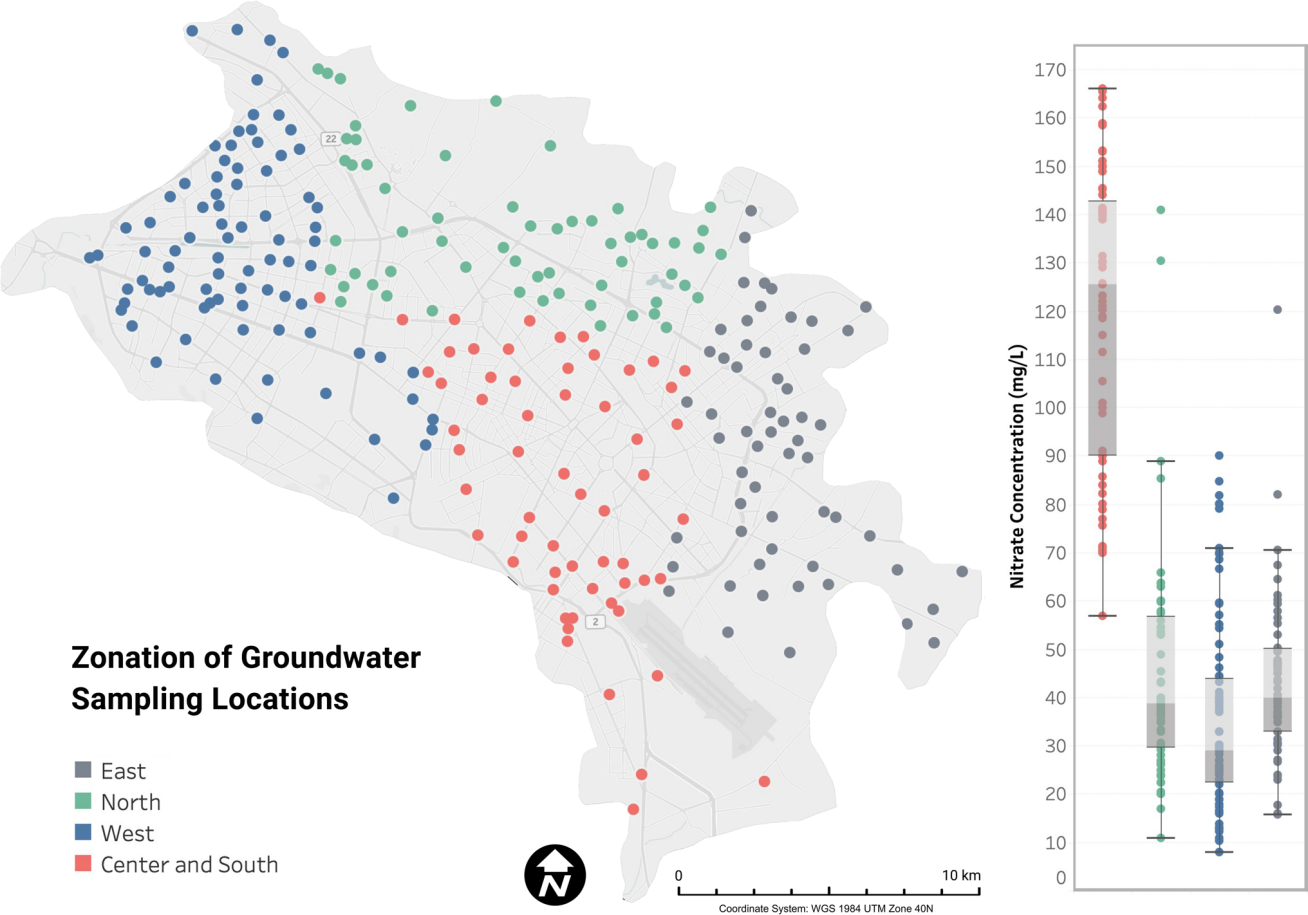
Different zones in the study area for the calculations of EDI of nitrate; green points represent samples from the northern sector, red points represent samples from central and southern sectors, blue and gray points represent samples from western and eastern parts, respectively. The boxplot shows the nitrate concentrations in each area

To calculate the EDI of nitrate from water, the city map was divided into four areas (i.e., south/central, north, west, and east), and the average concentration of nitrate was used separately in the calculations for each specified area. Figure 2 shows the zonation of the sampling locations. The average consumption rate of water per person was considered to be 2 L/day. The rate for vegetables and fruits for Iranian consumers was considered to be 286, and 142 g/day, respectively (Esteghamati et al. 2012; Sheikholeslam, 2001). We additionally computed the EDI (and Health Risk Index; HRI) if a person, according to the FAO and WHO guidelines, consumed 400 g of fruits and vegetables (FAO/WHO, 2005). In the calculations, the bodyweight of adult consumers and children was considered 70 kg and 31 kg, respectively (Hosseini et al. 1998). For the evaluation of health risks associated with the consumption of nitrate-containing water, vegetables, and fruits, the calculated EDI was subsequently compared with the ADI.

With a value of HRI less than 1 for any nitrate in water and food items, the consumer population is assumed to be safe and not exposed to the associated health hazards (USEPA, 2004). The HRI can be determined by the food item’s EDI value together with the oral Reference Dose (Rfd). The oral RfD is an estimation of the daily exposure of the human population, including sensitive subgroups (e.g., children), to dietary nitrate over the course of a lifetime without the significant risk of harmful effects (Barnes et al. 1988). The HRI for exposure to nitrate through consumption was determined applying Equation (2), recommended by earlier studies (Abtahi et al. 2018; Rahmani et al. 2018).

2 $$HRI= EDI/ Rfd$$

In Equation (2), EDI is in mg/kg body weight/day (expressed as nitrate ion), and Rfd is expressed in mg/kg body weight/day. According to the United States Environmental Protection Agency (USEPA), the oral Rfd of the nitrate-nitrogen is determined to be 1.6 mg/kg body weight/day, which is equivalent to 7.09 mg/kg body weight/day of nitrate (USEPA, 2013).

### Statistical analysis

Data processing for groundwater nitrate was conducted using IBM SPSS software v25. The spatial variability of the groundwater nitrate concentrations was conducted by interpolating sampling points applying the algorithmic method ‘Inverse Distance Weighted’ (IDW) integrated into the ArcGIS 10.5 software package.

For nitrate exposure evaluations, the normal distribution of nitrate data and EDI values was determined by applying Shapiro–Wilk test. Due to the not-normally distributed data, the relative nitrate content of different groups was analyzed using the Kruskal–Wallis test, and for the comparison of each group, the Bonferroni correction was applied. The statistical significance level was adjusted as *p* < 0.05. The analyses were executed using SPSS software (version 28.0, IBM SPSS, Chicago, IL, USA). For categorizing tested samples with regard to their nitrate content, Ward’s hierarchical cluster analysis was applied, using RStudio for macOS (version 1.4.1717).

## Results and discussion

### Nitrate concentration in groundwater

**Fig. 3:**
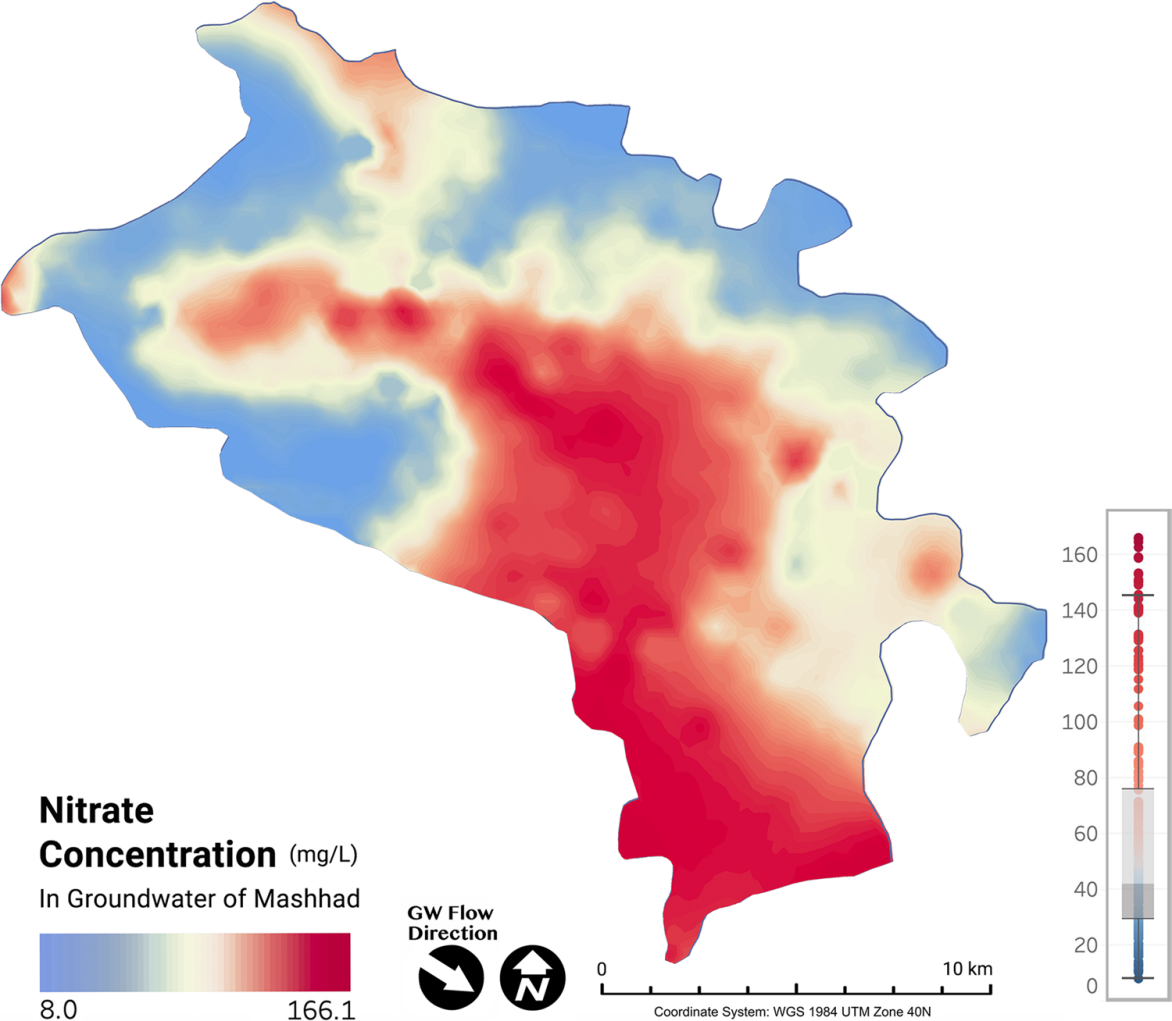
Spatial distribution of groundwater nitrate concentration in the study area

The concentration of nitrate in the groundwater of the Mashhad urban area is illustrated in Fig. 3, which ranges from 8 to 166.1 mg/L. Nitrate concentrations exceeded the prescribed permissible limits of 50 mg/L (World Health Organization, 2017) for nitrate in drinking water in 42% of the wells. Central and southern parts of the city demonstrate elevated nitrate concentrations (with mean concentration of 119.37 mg/L), whereas the western area shows a significantly lower concentration of nitrate (mean concentration of 35.69 mg/L). The maximum concentration value of nitrate (166.1 mg/L) belongs to the study area’s central and southern parts, representing higher population density. In contrast, the minimum value (8 mg/L) was obtained from newly constructed and less populated western areas (boxplot in Fig. 2). There is no known lithological source for this pollutant, indicating that nitrate mainly originates from anthropogenic sources (Ritzi et al. 1993; Marie and Vengosh, 2001). An earlier study has shown strong correlations between nitrate and other co-migrant ions (i.e., chloride, phosphate, and sulfate), indicating that these pollutants have arisen from the same origin of the sewage leakage into the groundwater of Mashhad. This anthropogenic source of nitrate contamination was also reconfirmed using nitrate isotopic analysis (Zendehbad et al. 2019a). While the western parts have already been equipped with the new sewage collection systems, completion of the construction of such systems in the central and southern parts can help mitigate the nitrate contamination and minimize anthropogenic impacts on the city’s drinking water source.

### Coliform and turbidity

Coliform and turbidity tests were also conducted on the above samples as they are indicators of the sanitary quality of water. The presence of total coliform in the drinking water can be a sign of the contamination of water with urban and human wastes due to effluent from septic systems and untreated sewage discharge, which can contribute amounts considerably above those naturally present in groundwater (Lang et al. 2006; Marshall et al. 2019). Drinking water samples should have zero total coliform count per 100 mL, according to the Institute of Standards and Industrial Research of Iran (ISIRI) and WHO (World Health Organization, 2017). The high amount of coliforms in groundwater may induce intestinal diseases, including typhoid, hepatitis A and E, dysentery, cryptosporidiosis, and diarrhea (Ling, 2000).

**Fig. 4:**
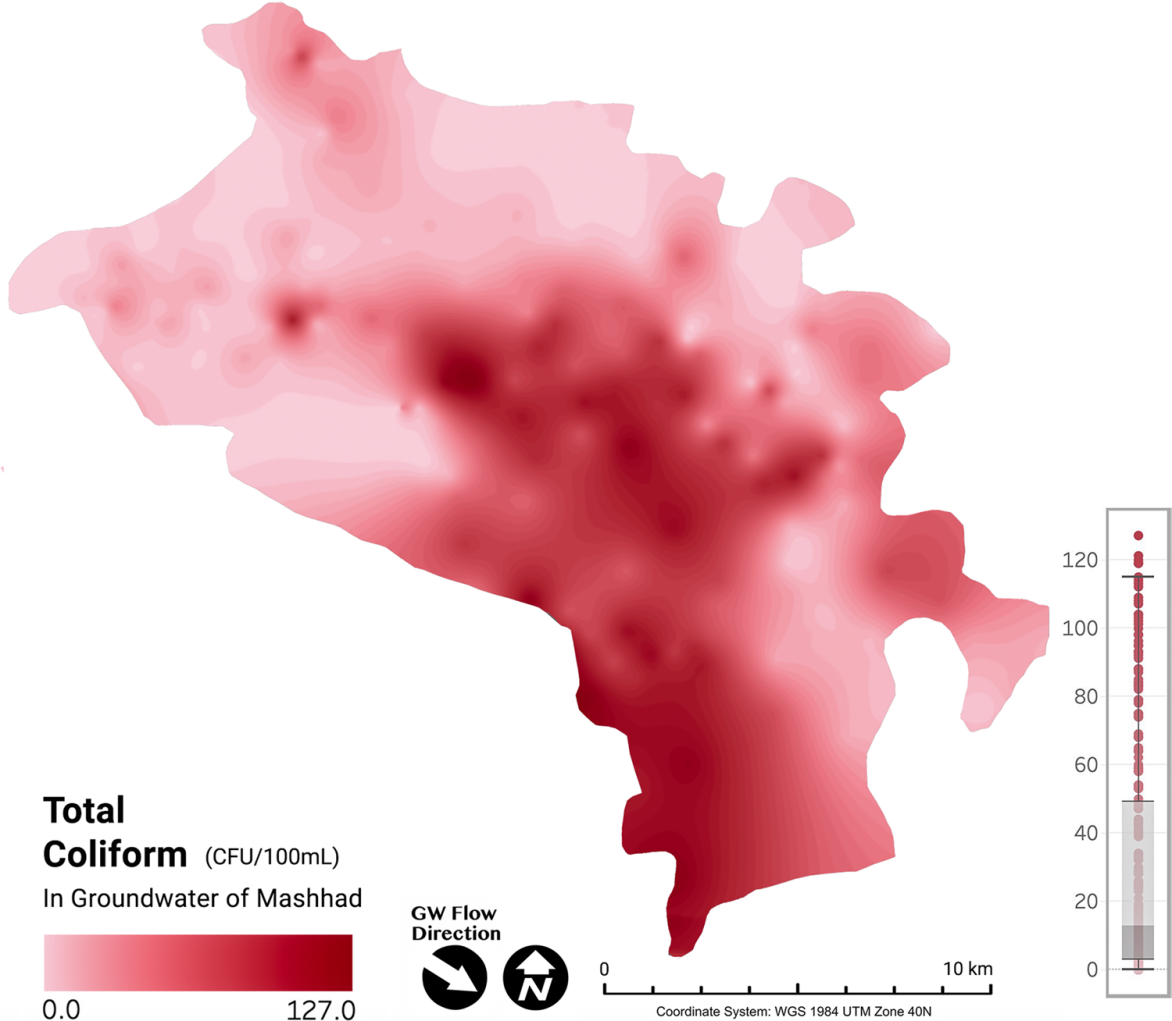
Spatial distribution of total coliform concentration in the groundwater of the study area

The present study witnessed the total coliform value ranging from 0 to 127 CFU/100 mL (55 wells < 1, including 32 wells = 0 CFU/100 mL). Total coliforms are violating the prescribed limit at 78.93% of the sampling locations. As with nitrate, total coliforms were generally much higher over the city’s central and southern areas. Figure 4 shows that the most affected parts by nitrate pollution from septic systems have also the highest total coliform concentrations. The groundwater is affected due to the insufficient sewer system and household waste infiltration caused by septic or adsorption wells, mainly in older areas of the city.

**Fig. 5:**
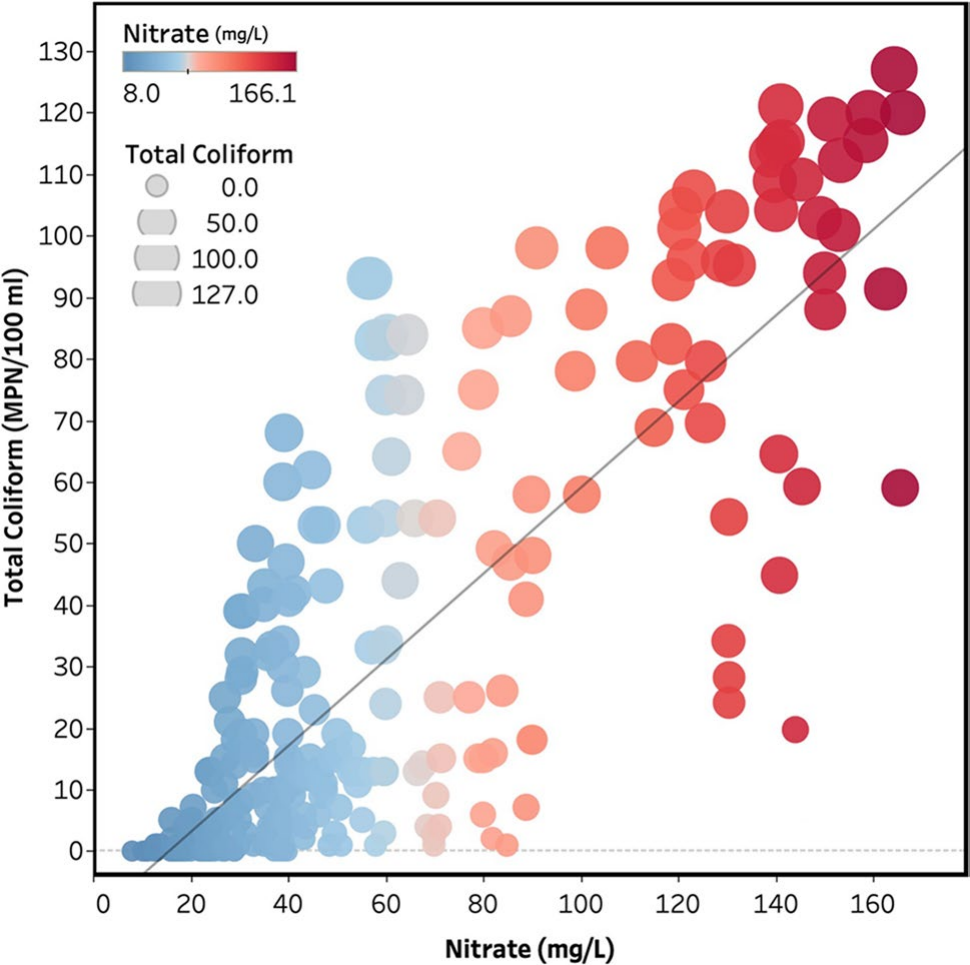
Linear relationship between total coliform and nitrate concentrations in groundwater of Mashhad

Furthermore, concentrations of nitrate and total coliform counts confirmed relatively strong correlations (*R*^2^ = 0.63), specifying that they have arisen from the same origins (Fig. 5). Hence, it can be concluded that nitrates are released in the system by anthropogenic sources (i.e., manure and sewage), which is consistent with the previous study’s findings (Zendehbad et al. 2019a). Based on the data, it would appear that nitrate measurements in the locations affected by a single sewage source of contamination can be a good indicator of coliform and bacterial contamination levels.

Turbidity, a measure of the light refractiveness of water, is an evaluation of the degree to which water loses its transparency due to the presence of suspended matters, either organic or inorganic, chemical or biological particulates. Leaching of industrial and domestic wastes can contribute to turbidity in groundwater samples. The turbidity in the water samples is an indication of water pollution, particularly due to the source near the adsorbing wells or cesspools. Inorganic nutrients such as nitrogen and phosphorus present in agricultural runoff stimulate the growth of algae, which also contributes to turbidity. There are chances for the pathogenic organisms to be enclosed in the turbidity-causing particles, thus leading to health hazards (Prakash and Somashekar, 2006). Turbidity can protect pathogenic microorganisms from the effects of the disinfection process and thus stimulate the growth of bacteria during storage. As presented in Table 2, our samples have turbidity ranging from 0 to 29 NTU. The majority (74.32%) of turbidity values are below the permissible limit of 5 NTU.

**Table 2:** Measured nitrate, total coliform, and turbidity in groundwater samples collected from Mashhad

Parameter	Min	Median	Max
Nitrate (mg/L)	8	42	166.1
Total coliform (CFU/100 mL)	0	13	127
Turbidity (NTU)	0	4	29

### Nitrate concentration in fruits and vegetables

**Fig. 6:**
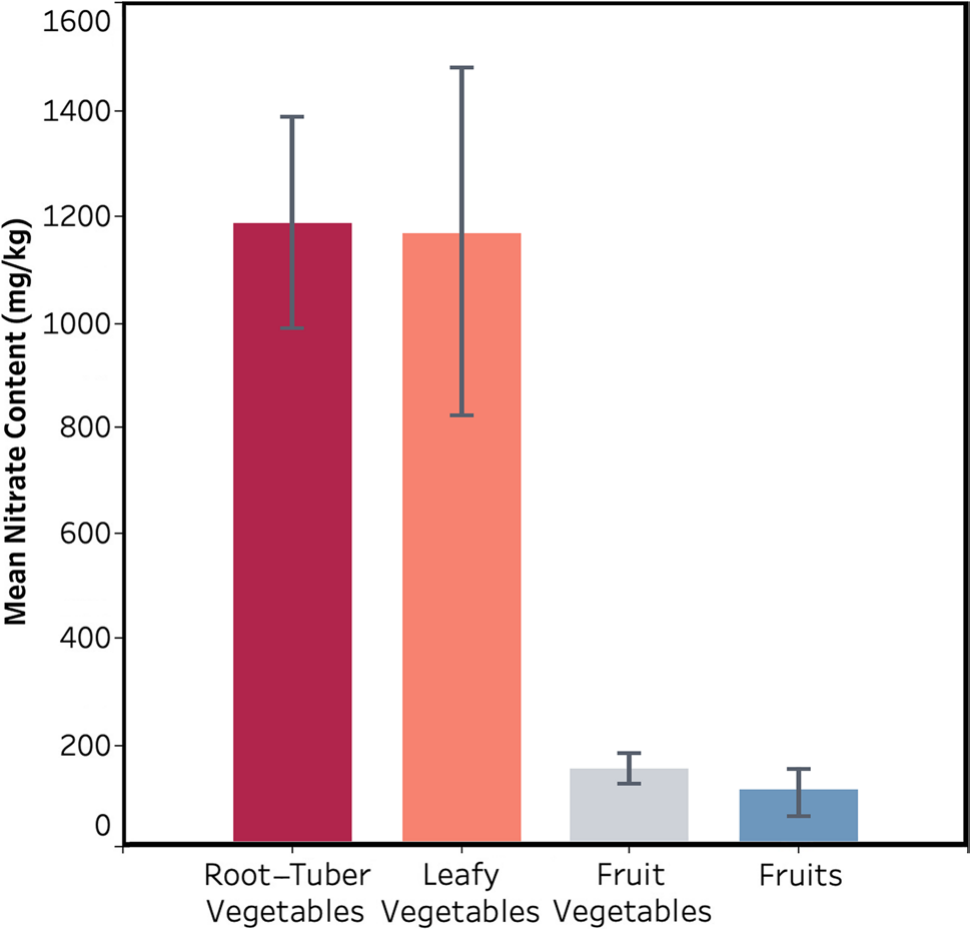
Mean nitrate concentrations in tested root and tuber vegetables, leafy vegetables, fruit vegetables, and fruit samples. The error bars express the standard deviations.

Vegetables and fruits are known to contribute to bringing a substantial amount of nitrates to the population’s diet. The samples tested in this work were exhibited with their respective nitrate concentrations and categorized in the earlier-mentioned categories of four. The nitrate concentration of the samples was detected, and the findings exhibit a substantial difference between the specified groups. As seen in Fig. 6, a significantly higher nitrate content was found in tuber and root vegetables (1193 mg/kg) and leafy vegetables (1173 mg/L) compared to fruit vegetables (160.13 mg/kg). Also, when compared to vegetables, the fruit samples were found to have a lower nitrate content (102.37 mg/kg).

**Fig. 7:**
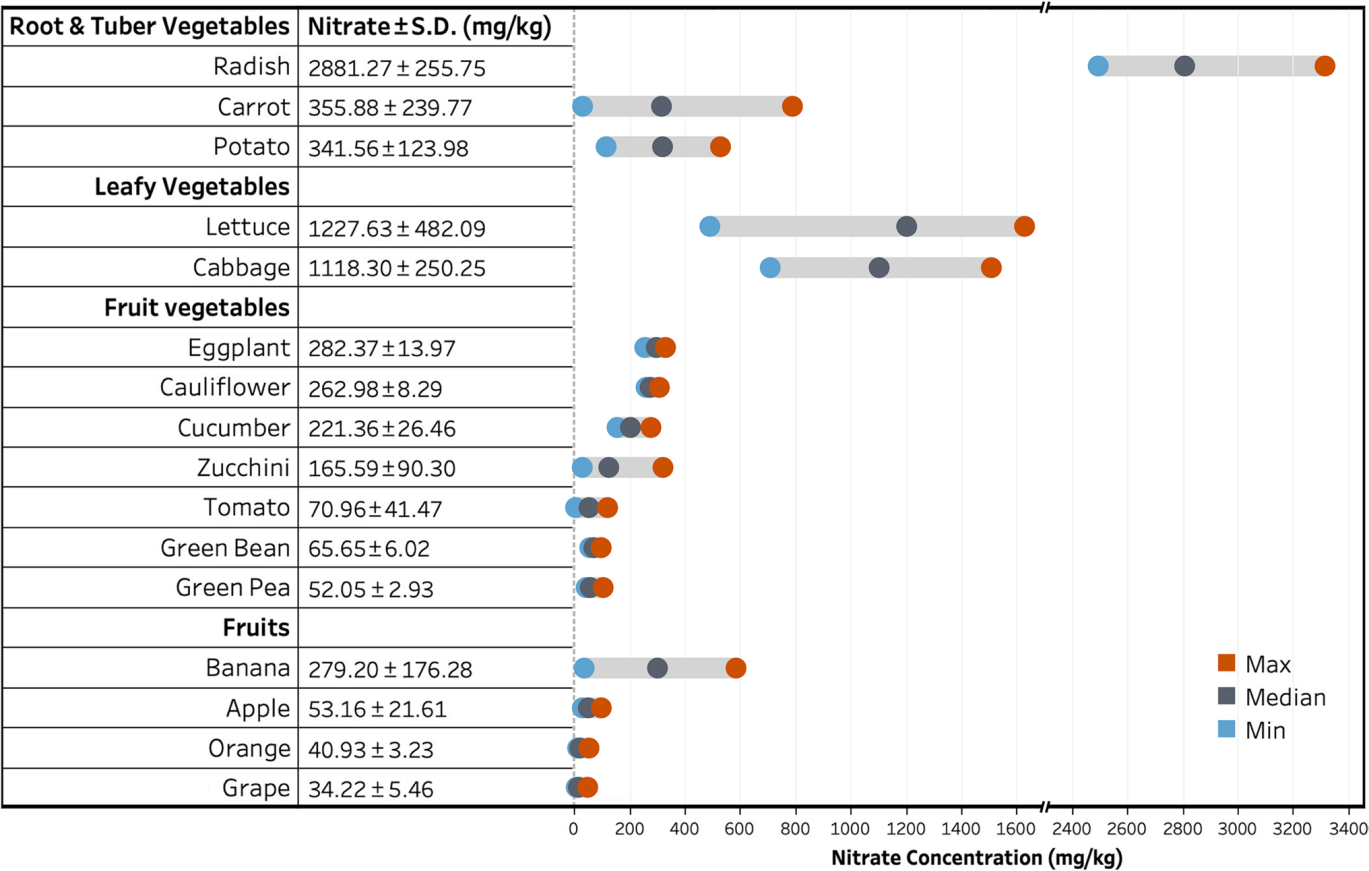
Mean nitrate concentrations in tested fruits and vegetables

As summarized in Fig. 7, among the individual type of root and tuber vegetables, radish demonstrates the highest mean nitrate concentration (2881.27 mg/kg), followed by carrot (355.88 mg/kg) and potato (341.56 mg/kg). The leafy vegetables of lettuce and cabbage were measured to have high nitrate contents (1227.63 and 1118.30 mg/kg, respectively). Among fruit vegetables, the highest nitrate content was determined in eggplant, and cauliflower (>250 mg/kg), followed by cucumber and zucchini (150–250 mg/kg). Other fruit vegetables have been observed to have a nitrate content of below 100 mg/kg. Among fruits, the highest nitrate content appears in bananas (279 mg/kg), while other fruits of apple, orange, and grape show mean nitrate concentration of below 45 mg/kg.

**Fig. 8:**
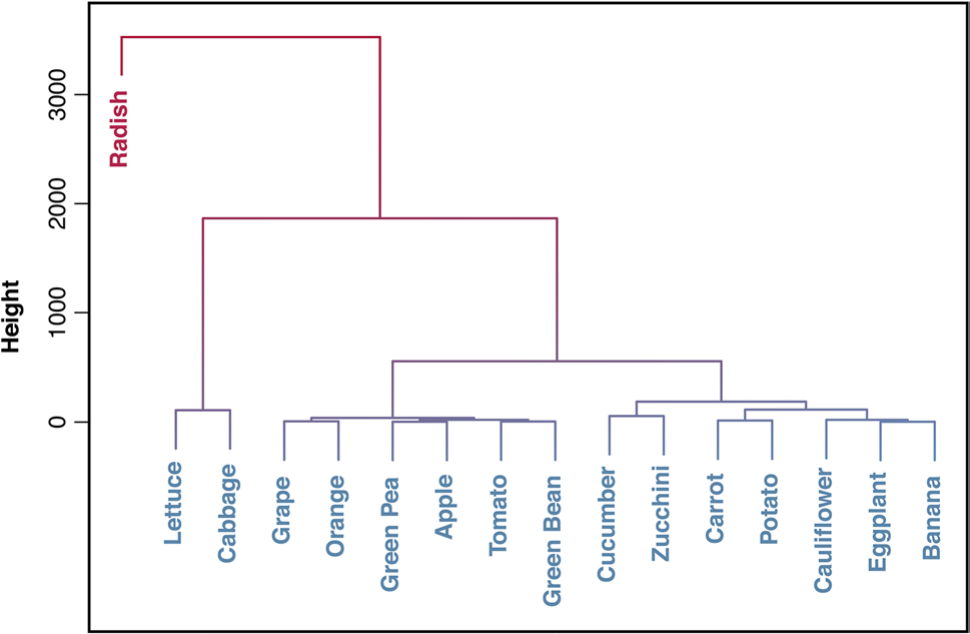
Dendrogram displaying hierarchical clustering for fruits and vegetables’ nitrate contents

Figure 8 shows the hierarchical clustering for nitrate accumulation in fruits and vegetables, where the samples are grouped into four clusters. The first cluster consists of banana, eggplant, cauliflower, potato, carrot, zucchini and cucumber; this group is accountable for 6.2% of the total nitrate content. Fruits such as apple, orange, and grape as well as green beans, green pea and tomato comprise the second cluster, contributing to 1.21% of the total nitrate content. The third cluster, which contributes to 26.79% of the total nitrate content, includes leafy vegetables of lettuce, and cabbage. The fourth cluster is only comprised of radish by contributing to 65.8% of the total nitrate content.

The findings suggest that root and tuber vegetables, and leafy vegetables have the highest nitrate levels, followed by fruit vegetables, and then fruits. This order is in line with those of some other studies (Sušin et al. 2006; Bahadoran et al. 2016). The use of fewer fertilizers in fruit farming could be a reason for reduced nitrate contents in fruits.

The mean nitrate concentrations detected in fruits and vegetables (mg/kg) in Mashhad clearly demonstrate lower values compared to the reported values from other studies (De Martin and Restani, 2003; Feng et al. 2006; Temme et al. 2011; Bahadoran et al. 2016; Stavroulakis et al. 2018; Roila et al. 2018). The concentrations of nitrate in the eggplant, cauliflower, zucchini, green bean and green pea were found to be comparable to the other Iranian studies’ findings (Bahadoran et al. 2016; Mehri et al. 2019; Taghipour et al. 2019). As summarized in Table 3, the vegetable nitrate contents of this study are comparable to the reported values from other countries such as Japan, UK, and Italy. Previous studies have linked lower nitrate concentrations in some fruits and vegetables to longer durations of sunshine and higher temperatures (Escobar-Gutierrez et al. 2002). Mashhad climate is also characterized by high temperatures in summer along with long periods of sunshine. Such contributing factors could be an explanation for the overall lower nitrate levels found in present research, particularly in fruits. On the other hand, high nitrate levels and relatively high variability between the individual types of fruit and vegetable samples, which lead to some samples’ large standard deviation, might be attributable to the soil type, application of fertilizers, agricultural practices, time of harvesting and groundwater nitrate contamination (Amr and Hadidi, 2001; Mahvi et al. 2005; Abdulrazak et al. 2014). However, in this regard, further investigation is required.

**Table 3:** The reported nitrate contents for commonly consumed vegetables in different countries

Items	Nitrate content (mg/kg)										
	Germany (Reinik et al. 1990)	Italy (De Martin and Restani, 2003; Santamaria et al. 1999)	UK (Meah et al. 1994; Ysart et al. 1999)	France (Menard et al. 2008)	USA (Keeton et al. 2009)	Tunisia (Razgallah et al. 2016)	Turkey (Ayaz et al. 2007; Mor et al. 2010)	Korea (Chung et al. 2003; Reinik et al. 1990)	China (Chung et al. 2011; Zhou et al. 2000)	Japan (Himeno et al. 2003; Reinik et al. 1990)	Present study
Lettuce	750–5500	672–1745	1051–2330	1973–5600	90–1400	795–1200	914–1439	33–3944	340–2700	1226	1227.63
Cabbage	3100	400–3270	26–1523	498–1855	418–2700	1842–2160	510	222–1740	480–2900	1040–1100	1118.30
Carrot	–	28–394	11–566	120–861	ND–2800	440–1800	190	ND–1005	43–490	193–1031	355.88
Radish	780–2400	1117–2993	2600	1860–5000	60–9000	1800–2800	3428	ND–3486	414–2078	1060–2725	2881.27
Potato	–	81–179	75–283	191–730	57–1000	215–1080	–	ND–640	100–346	199–713	341.56
Tomato	–	50.0	4–42	17.6–157	ND–170	25.0–91.0	ND–71.0	–	1–180	1.55	70.96
Cucumber	–	–	–	191–1800	17–570	800–959	–	–	–	–	221.36

### Health risk assessment

The degree of nitrate toxicity is directly proportional to the amount consumed on a daily basis. In this study, the estimated EDI and HRI of nitrate for water, fruits, and vegetables were computed using Equations (1) and (2). The results are shown in Table 4. While groundwater of the south and central part of the city shows the highest EDI value of nitrate, the western region has the lowest, followed by eastern and northern parts. The south and central parts’ EDI value for adults is marginally within the acceptable limit; however, children’s estimated daily nitrate intake from drinking water of these regions is more than 7 mg/kg body weight, far higher than the prescribed limit. The ADI for children in the area is determined to be higher than the maximum allowable ADI limit (3.7 mg/kg body weight/day: WHO). These regions’ HRI value is also above the acceptable level of 1 for the children target group. Therefore, based on the calculations, it could be inferred that potentially there is no significant health risk from nitrate through the daily intake of drinking water in the children and adult population in the west, east, and north. In contrast, the potential health risks of nitrate intake can be assumed for children in the south and central Mashhad, given that they consume more water per kilogram of body weight. In similar studies in the south and east of Iran, children were reported to be more significantly exposed to the health risks of drinking water contaminants compared to adults (Rezaei et al. 2017; Qasemi et al. 2018). Likewise, the health risks associated with groundwater consumption in other parts of the world, such as China, were found to be more significant in children in comparison to adult consumers by having a higher risk index for infants and children (Su et al. 2013; Chen et al. 2017). The deterioration of water quality by the injection of nitrate to the groundwater through inefficient sewer systems can be the reason for getting relatively higher HRI and EDI values in the south and central Mashhad.

**Table 4:** EDI and HRI for nitrate in drinking water according to the current consumption rate, and for the fruits and vegetables in accordance with both current daily consumption rate and FAO/WHO recommendation for children and adults

Items	Based on current rate of consumption ^a^						Based on WHO-recommended consumption rate ^b^					
	EDI ^c^		%ADI		HRI		EDI ^c^		%ADI		HRI	
	Adult	Children	Adult	Children	Adult	Children	Adult	Children	Adult	Children	Adult	Children
Groundwater												
North	1.26	2.84	34.04	76.85	0.18	0.40						
South and Central	3.41	**7.70**	92.18	–	0.48	**1.09**						
East	1.23	2.78	33.30	75.19	0.17	0.39						
West	0.98	2.21	26.44	59.69	0.14	0.31						
Leafy vegetables												
Lettuce	**5.02**	**11.33**	–	–	0.71	**1.60**	**7.02**	**15.84**	–	–	0.99	**2.23**
Cabbage	**4.57**	**10.32**	–	–	0.64	**1.46**	**6.39**	**14.43**	–	–	0.90	**2.04**
Root and tuber vegetables												
Carrot	1.45	3.28	39.30	88.74	0.21	0.46	2.03	**4.59**	54.96	–	0.29	0.65
Radish	**11.77**	**26.58**	–	–	**1.66**	**3.75**	**16.46**	**37.18**	–	–	**2.32**	**5.24**
Potato	1.40	3.15	37.72	85.17	0.20	0.44	1.95	4.41	52.75	–	0.28	0.62
Fruit vegetables												
Tomato	0.29	0.65	7.84	17.69	0.04	0.09	0.41	0.92	10.96	24.75	0.06	0.13
Cauliflower	1.07	2.43	29.04	65.57	0.15	0.34	1.50	3.39	40.61	91.71	0.21	0.48
Green Bean	0.27	0.61	7.25	16.37	0.04	0.09	0.38	0.85	10.14	22.89	0.05	0.12
Zucchini	0.68	1.53	18.29	41.29	0.10	0.22	0.95	2.14	25.57	57.75	0.13	0.30
Cucumber	0.90	2.04	24.44	55.20	0.13	0.29	1.26	2.86	34.19	77.20	0.18	0.40
Eggplant	1.15	2.61	31.18	70.41	0.16	0.37	1.61	3.64	43.61	98.47	0.23	0.51
Green Pea	0.21	0.48	5.75	12.98	0.03	0.07	0.30	0.67	8.04	18.15	0.04	0.09
Fruits												
Apple	0.11	0.24	2.91	6.58	0.02	0.03	0.30	0.69	8.21	18.54	0.04	0.10
Banana	0.57	1.28	15.31	34.57	0.08	0.18	1.60	3.60	43.12	97.37	0.23	0.51
Orange	0.08	0.19	2.24	5.07	0.01	0.03	0.23	0.53	6.32	14.27	0.03	0.07
Grape	0.07	0.16	1.88	4.24	0.01	0.02	0.20	0.44	5.28	11.93	0.03	0.06

^a^For vegetables and fruits: 286, and 142 g/day, respectively; For water: 2 L/day

^b^For vegetables and fruits: 400 g/day

^c^mg NO_3_^−^/kg body weight/day

Values expressed in bold exceed the acceptable limits

Among the individual type of root and tuber vegetables, the highest nitrate EDI is found in radish for consumers, followed by carrot and potato, based on both current and WHO-recommended consumption rates. Likewise, leafy vegetables of lettuce and cabbage also exceeded the EDI limits. Only in the case of radish, lettuce, and cabbage does the HRI exceed the acceptable level of 1 for children and adults. Adults’ daily nitrate intake from fruit vegetables such as eggplant, cauliflower, and cucumber is estimated to be approximately 1 mg/kg body weight (expressed as nitrate ion), whereas children’s intake appears to be around 2 mg/kg body weight. If the consumption considers being according to the WHO recommendation, the value for children ranges from ~3 to 4 mg/kg body weight (expressed as nitrate ion). Adults’ current estimated daily intake of nitrate from other fruit vegetables is less than 1 mg/kg body weight, except for bananas. In bananas, the EDI ranges from 0.5 to 3.5 mg/kg body weight for adults and children. Additionally, the HRI for all of the fruit vegetables, and fruits analyzed is less than 1, which is within the acceptable range.

The results obtained in our study for the EDI of cauliflower, cucumber, carrot, tomato, and potato appeared to be higher compared to the values reported in a study from Poland (0.03, 0.16, 0.03, 0.04, 0.84 mg NO_3_^−^/kg body weight, respectively) (Gruszecka-Kosowska and Baran, 2017). More similar to our results, the values of EDI for tomato (0.17 mg NO_3_^−^/kg body weight) were reported by Mehri et al. (2019). However, our obtained EDI values appear to be higher for potato and carrot samples than those reported in their study (0.13 and 0.19 mg NO_3_^−^/kg body weight, respectively).

Radish, lettuce and cabbage were detected to exceed the acceptable limit and potentially pose risks for both children and adult consumers, when taking the value of 3.7 mg NO_3_^−^/kg body weight/day as the benchmark of ADI threshold (Hambridge, 2003); and HRI < 1 as the HRI threshold benchmark. Conversely, several studies have reported that none of their samples exceeds the standard ADI value (Suh et al. 2013; Gruszecka-Kosowska and Baran, 2017; Sebaei and Refai, 2021). Nitrate-contaminated irrigation water usage and fertilization intensity could be a reason why radish, lettuce, and cabbage samples exceed the threshold ADI limit in our study.

The health risk index and estimated daily intake for each fruit and vegetable sample were determined based on the 400 g daily consumption rate. Accordingly, radish, lettuce, and cabbage for adult consumers and radish, lettuce, cabbage, carrot, and potato for children were found to exceed the maximum allowable ADI limit (3.7 mg/kg body weight/day: WHO). Whereas, in the case of HRI, only radish, lettuce, and cabbage exceeded the permissible limit. Therefore, the present study concludes that nitrate pollution’s adverse effects in fruits and vegetables in Mashhad are relatively tolerable. However, the lower values of EDI and HRI could be in connection with a lower than the recommended norm of consumption for fruits and vegetables (400 g/person/day: WHO, 2008) as well as lower nitrate contamination of fruits and vegetables.

It is important to take into account that drinking water, fruits and vegetables consumptions are not considered the only (but major) exposure ways to nitrate and that an HRI value below 1 cannot solely reflect a healthy and safe level of nitrate intake. Other potential ways of exposure, including meat and other food products, are beneficial in further investigating the health risks of nitrate intake.

In our study, several commonly consumed fruit and vegetable samples available during our research period were investigated for their nitrate levels. To present a broader picture of nitrate content in fruits and vegetables in Mashhad, further research with a larger sample size is also recommended.

## Conclusion

The major concerns regarding human exposure to nitrate through a nitrate-rich diet are the endogenous generation of carcinogenic nitrosamines. Drinking water, fruits, and vegetables are regarded as the primary contributing factors to dietary nitrate. The present study shows that the mean nitrate concentration in the groundwater of the west, north, and east of the Mashhad city, as well as the majority of fruit and vegetable samples, were found to be comparatively lower than that of the standard threshold. The maximum concentration value of nitrate (166.1 mg/L) belonged to the study area’s central and southern parts, representing higher population density. In contrast, the minimum value (8 mg/L) was obtained from newly constructed and less populated western areas. Increased levels of nitrate in some fruits and vegetables can be attributable to prolonged and ineffective storage and excessive application of chemical fertilizers along with nitrate-contaminated irrigation water. Significantly higher nitrate content was found in the root and tuber vegetables (1193 mg/kg) and leafy vegetables (1173 mg/L) compared to fruit vegetables (160.13 mg/kg). Also, when compared to vegetables, the fruit samples were found to have a lower nitrate content (102.37 mg/kg). With the exception of radish, lettuce, and cabbage, as well as south and central groundwater of Mashhad, the values of HRI for nitrate in all the other samples were calculated to be less than 1, which signifies that the associated health risks with nitrate exposure can be considered not significant. Hence, the nitrate intake through the majority of groundwater and vegetables and all the fruits analyzed in this study can be regarded as safe for consumers.

Concerning water quality, the coliform and nitrate levels are significant factors constraining the suitability of groundwater for human consumption in the area. Confirming with relatively strong correlations (*R*^2^ = 0.63), the high total coliform found in most samples was linked to the concentrations of nitrate, and both are likely derived from infiltration of household wastes through septic or adsorption wells, particularly in the old south and central parts. Total coliforms violated the prescribed limit at 78.93% of the sampling locations, ranging from 0 to 127 CFU/100 mL (55 wells < 1, including 32 wells = 0 CFU/100 mL). The majority (74.32%) of turbidity values were also found to be below the permissible limit.

The findings of our study are expected to provide a better picture of the residents’ exposure to nitrate through different routes (i.e., water and agricultural products) by quantifying their nitrate levels, combining both routes’ data, and assessing the associated health risks in Mashhad, in order to assist public health providers and governments’ regulators in developing better-detailed management plans.

## Data Availability

The datasets used and/or analyzed during the current study are available from the corresponding author on reasonable request.
